# Mobile Code Anti-Reversing Scheme Based on Bytecode Trapping in ART

**DOI:** 10.3390/s19112625

**Published:** 2019-06-10

**Authors:** Geonbae Na, Jongsu Lim, Sunjun Lee, Jeong Hyun Yi

**Affiliations:** 1 School of Computer Science and Engineering, Soongsil University, Seoul 06978, Korea; nagb@ssu.ac.kr (G.N.); jongsu253@ssu.ac.kr (J.L.); 2 School of Software, Soongsil University, Seoul 06978, Korea; starj1024@soongsil.ac.kr

**Keywords:** internet of things, reverse engineering, Dalvik, ART, mobile code protection

## Abstract

As interest in Internet of Things environments rapidly increases throughout the IT convergence field, compatibility with mobile devices must be provided to enable personalized services. The security of mobile platforms and applications is critical because security vulnerabilities of mobile devices can be spread to all things in these environments. Android, the leading open mobile platform, has long used the Dalvik virtual machine as its runtime system. However, it has recently been completely replaced by a new runtime system, namely Android Runtime (ART). The change from Android’s Dalvik to ART means that the existing Dalvik bytecode-based application execution structure has been changed to a machine code-based application execution structure. Consequently, a detailed understanding of ART, such as new file formats and execution switching methods between codes, is required from the viewpoint of application security. In this paper, we demonstrate that an existing Dalvik-based application vulnerability can be exploited as-is in ART. This is because existing Dalvik executable files coexist in the ART executable file, and these Dalvik bytecodes and compiled machine codes have *one-to-one mapping* relationships. We then propose an ART-based application protection scheme to secure this by dynamically eliminating the one-to-one mapping. In addition, the proposed scheme is implemented to evaluate its reverse engineering resistance and performance through experiments.

## 1. Introduction

With the proliferation of mobile devices such as smartphone, the use of smart sensors has increased, convergence and connectivity between devices have been secured, and interest in the Internet of Things (IoT) environment has rapidly increased throughout the IT convergence field. ICBM (IoT, Cloud, Big Data, and Mobile), which is currently the most important issue in the IT industry, is attracting attention as the next-generation growth engine. Application of the IoT to reality from an Internet-based convergence center is expected to increase efficiency and convenience and diversify economic values. However, to achieve such a positive outcome in the future, it is necessary to solve various risk factors implied by the IoT. For example, to connect various devices, compatibility problems between devices should be solved. Furthermore, to provide personalized services, the compatibility of mobile devices with intensive personal information will inevitably become necessary [1].

The largest share of the mobile platform market is occupied by Google Android. The number of IoT devices that are compatible with Android is projected to reach over 25 billion by 2021 according to Gartner [2]. In this hyper-connected service environment, mobile devices are vulnerable to security threats that can infect all connected IoT devices. This means that we live in an era where the security of mobile platforms is of the utmost importance. Android, as a typical open mobile platform, has been experiencing various security problems due to the Dalvik-based self-signing application structure [3]. Android applications implemented in the Java language and distributed as Android application package (APK) files can be easily restored as smali code or original Java source code using reversing tools such as apktool [4] or dex2jar [5]. The code of the disassembled (or decompiled) application is easily exposed to attackers. As an attacker can analyze the code, the core application code can be bypassed or modified, which can cause serious issues for the application developer. Furthermore, malicious code can be inserted into the application, which is then redeployed as a benign application, thereby extending such issues and damage to general users [6,7].

Several protection techniques have been explored in a variety of areas, such as application code obfuscation [8], API hiding [9], tamper detection [10,11], and packing [12,13] to protect applications from these malicious behaviors. However, as described above, these protection techniques and tools [14,15,16] are not safe from attackers owing to the structural characteristics of Android, in which it is relatively easy to analyze the code [17,18,19,20,21]. Therefore, researchers are reinforcing the complement through continuous improvement of vulnerability, and attackers continue to analyze and utilize these technologies.

In terms of runtime systems, Android Runtime (ART), a new runtime system for Android [22], has emerged and completely replaced Dalvik VM in Android 5.0. ART runs applications through direct machine code, unlike the existing Dalvik VM-based runtime system, which does so by running the application’s Dalvik bytecode through the interpreter. There are several improvements in ART compared with the existing Dalvik VM, including improved performance of the application’s machine code without the need for an interpreter. However, the reversing vulnerability due to code exposure in the existing Dalvik VM has not been clearly solved, and the present reverse engineering analysis technique can still be applied to the newly introduced ART [23,24,25,26,27].

Essentially, this is because existing Dalvik Executable (DEX) files coexist in the Optimized Ahead-of-Time (OAT) file [28], which is the ART executable file, and these DEX and compiled machine codes have *one-to-one mapping* relationships. For this reason, if it can be artificially manipulated to call the DEX file inside the OAT, the vulnerability of the existing Dalvik VM can be exploited as-is in ART. Therefore, in this paper, we propose a scheme to overcome this vulnerability by eliminating the one-to-one mapping relationship between bytecode and machine code and exposing the disguised bytecode to confuse analysts.

This paper is organized as follows. Section 2 is an analysis of ART, the latest Android runtime system. Section 3 presents a newly discovered security vulnerability based on the ART structural characteristics. Section 4 introduces the proposed scheme to solve the security vulnerability. Section 5 describes experiments with the implementation of the proposed scheme. Section 6 discusses the issues considered in the proposed scheme. Section 7 concludes the paper.

## 2. Background

ART was first introduced to Android 4.4 (KitKat). Since Android 5.0 (Lollipop), the Dalvik VM has been completely replaced by ART as the default runtime system [29]. In this section, we examine differences between Dalvik and ART from the viewpoints of file and execution structure.

### 2.1. Differences between Dalvik VM and ART

#### 2.1.1. Installation and Execution

Android applications are implemented in Java with high productivity and portability. The Android execution environment also has an execution structure very similar to Java. The only difference is that, for Android, the DEX file is distributed, which is the result of compiling the Java bytecode (intermediate language) with Dalvik bytecode using the dx tool. This distributed Android application runs on the Android device that contains the Dalvik VM.

Dalvik VM and ART differ largely in how they install and run applications internally. For the Dalvik VM, the dexopt tool creates an Optimized DEX (ODEX) file for the DEX file in the deployed APK at the time of application installation. The ODEX file is the actual executable file in the Dalvik VM environment and is almost the same as the original DEX file. However, some of the opcodes are optimized for the execution environment, or the file with Inline expansion is applied. On the other hand, in the case of ART, the DEX file is compiled through the dex2oat tool into a completely new form called an OAT file. The OAT file is the result of ART’s Ahead-Of-Time (AOT) compilation [30] and is a substantial executable file in ART that replaces the ODEX file.

The two runtime systems that create their own executable files, ODEX and OAT, at the time of application installation also differ in their application execution. The Dalvik VM runs applications based on their Dalvik bytecode. At present, Just-In-Time (JIT) compilation [31] is used, which contrasts with the AOT compilation of ART. On the other hand, ART basically executes the application based on its machine code and, in some cases, by alternating the two code areas through an execution conversion structure between Dalvik bytecode and machine code.

#### 2.1.2. Compilation

Both JIT compilation and AOT compilation improve the execution performance of the application by generating its bytecode as machine code, but the time at which the machine code is generated differs. JIT compilation, which has been applied from Android 2.2 Froyo, generates machine code at the time of application execution. On the other hand, AOT compilation, applied with the introduction of ART, generates machine code at the time of application installation. Compared to JIT compilation, the AOT compilation at the time of installation improves execution performance. Compared with the existing Dalvik VM-based Android, ART-based Android has increased application installation time, and the capacity of the OAT file is also increased compared with the ODEX file in Dalvik VM.

### 2.2. Runtime Environment Transition

#### 2.2.1. OAT File Structure

**Figure 1:**
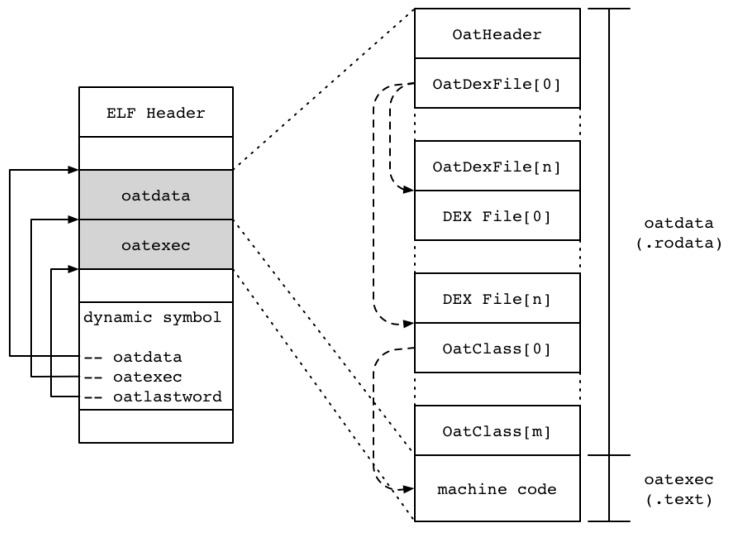
OAT file structure.

The OAT file is created through the dex2oat tool and follows the ELF file format, as shown on the left of Figure 1. This OAT file is an executable file in ART that consists of an oatdata section in the ELF file and an oatexec section. More specifically, there is a compiled machine code in the oatexec section of the OAT file. The oatdata section consists of OatHeader, OatDexFile, OatClass, etc. and also contains various information such as classes and methods to execute the application. There is a DEX file in this oatdata section for creating an ODEX file in an existing Dalvik VM. That is, the classes.dex file contained in the conventional APK (without special protection techniques such as changing the APK structure to prevent reversing) is still present. The inclusion of the DEX file in the OAT file creation process is closely related to the internal operation mechanism of ART to prepare for cases where the original machine code does not exist, such as an abstract method.

#### 2.2.2. Method Execution and Entry Point Management

Most methods in ART have a machine code compiled in the oatexec section in the OAT file, whereas Dalvik bytecode exists in the DEX file in the oatdata section of the OAT file. Most ART methods are based on compiled machine code, but certain methods operate through the interpreter as in existing Dalvik VM-s. For example, if the method is an abstract method, the machine code corresponding to the method does not exist, or if the runtime system is set to interpreter mode, it is executed as Dalvik bytecode instead of as its own machine code. In the case of abstract methods, the body of the method is defined in the child class, and so the machine code for the abstract method itself does not exist. Thus, abstract methods are always executed through the interpreter area.

Whether a method exists in machine code and whether the runtime system is in interpreter mode is determined by the NeedsInterpreter() function. If the machine code of the method does not exist, the entry point of the method is set such that the method is executed through Dalvik bytecode. Even if the machine code exists, the method is set to the interpreter mode based on the information of current_runtime_instance, and the execute method proceeds as Dalvik bytecode as described above.

**Figure 2:**
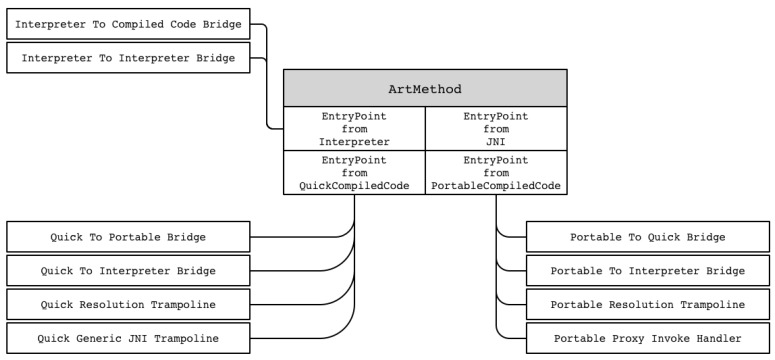
List of ArtMethod class entry points.

The execution transition mechanism caused by the mixture of machine code and Dalvik bytecode is performed by each method rather than by the application unit. Even if only a single application is run, the application methods will have their own execution flow. At the time the application is loaded for the method execution flow, the ArtMethod class sets the entry points for each method and changes the entry points during application execution. The ArtMethod class not only manages information such as method indexes and access flags, but also manages the entry point information of a method as a struct. Each entry points to one of several candidates are represented in Figure 2. For example, an Entry_Point_from_Interpreter points to either Interpreter_To_Interpreter_Bridge or Interpreter_To_Compiled_Code_Bridge.

The entry points of the method determine the entry point when called from each execution area, as the name suggests. The execution region is divided into an interpreter execution region for executing Dalvik bytecode and an execution region for executing the compiled machine code. For example, assuming that Method A calls Method B, if Method A is executed in the machine code execution area, the entry point of Method B would be Entry_Point_from_Quick_Compiled_Code, and if Method A is executed through the interpreter area, the entry point of Method B would be Entry_Point_from_Interpreter. Thus, in an environment where Dalvik bytecode and machine code executions are mixed, methods have multiple entry points. The actual execution switching is accomplished through the trampoline and bridge techniques described below.

#### 2.2.3. Bridge and Trampoline

**Figure 3:**
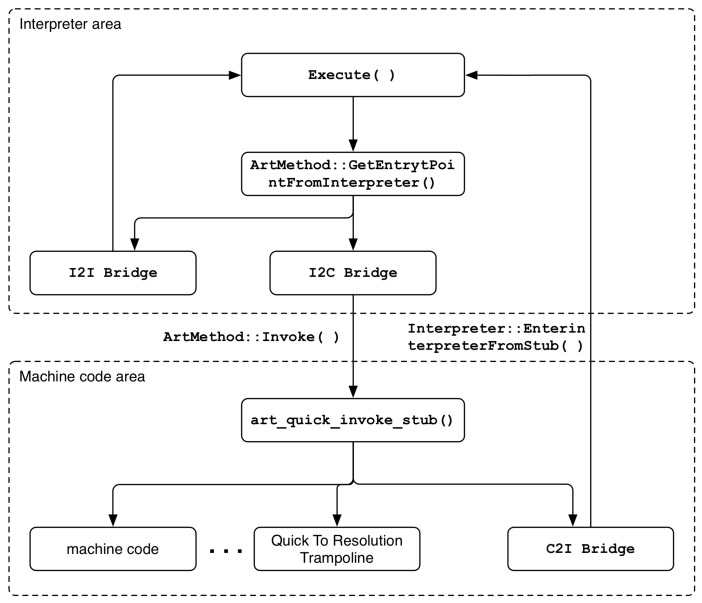
Execution switching between Interpreter region and machine code region.

The bridge is used to switch between the Dalvik bytecode execution region and the machine code execution region. The trampoline is used to find the address of the actual method to be called in order to support the dynamic loading and binding mechanism. Figure 3 shows the overall execution switching structure through bridge and trampoline.

Interpreter_To_Interpreter_Bridge (I2I Bridge): This bridge is used to maintain the execution flow from the interpreter area to the interpreter area. After calling the callee method in the call stack frame, it checks whether the class to which the method belongs is initialized, and finally executes the method through the Execute() function in the interpreter area.
Interpreter_To_Compiled_Code_Bridge (I2C Bridge): This bridge is called when switching the execution flow from the interpreter area to the machine code area. In the case of Interpreter_To_Interpreter_Bridge, the callee method is searched from the call stack frame to check whether the class to which the method belongs is initialized. However, the actual method is executed through the Invoke() function, which is a member method of the ArtMethod class, not the Execute() function. The Invoke() function internally calls the art_quick_invoke_stub() function, which is written in assembly language, to execute the machine code of the method. In other words, the Entry_Point_from_Quick_CompiledCode of the method is checked again, the entry set point is entered, and finally the machine code of the method is executed.
Quick_To_Interpreter_Bridge (C2I Bridge): This bridge is used when a transition from the compiled machine code execution region to the interpreter region is required. The bridge internally calls the EnterInterpreterFromStub() function, which causes the method to be executed in the interpreter area via the Execute() function, exactly like the final execution of Interpreter_To_Interpreter_Bridge described above.

## 3. Challenges to Reverse Engineering

### 3.1. Dynamic Debugging Support

In ART, the debugging mode uses Java Debug Wire Protocol (JDWP), similar to the one used in the existing Dalvik VM environment. In other words, in debugging mode, even though the compiled machine code of the target method exists, the Dalvik bytecode is executed through the interpreter area. Here, the target method indicates a method that has an event such as a break or watch when debugging. The debugging event is registered through the ProcessDeoptimizationRequest() function in the Dbg class and changes the entry point to the method of the executing application through the UpdateEntrypoints() function according to the request transmitted to the function.

Because of the use of the debugging mode to induce bytecode execution instead of the machine code in OAT, it is possible to apply the reverse engineering analysis technique that was originally operated in Dalvik. The following are a few best practices that allow an application to run in interpreter mode through a break event.

**Figure 4:**
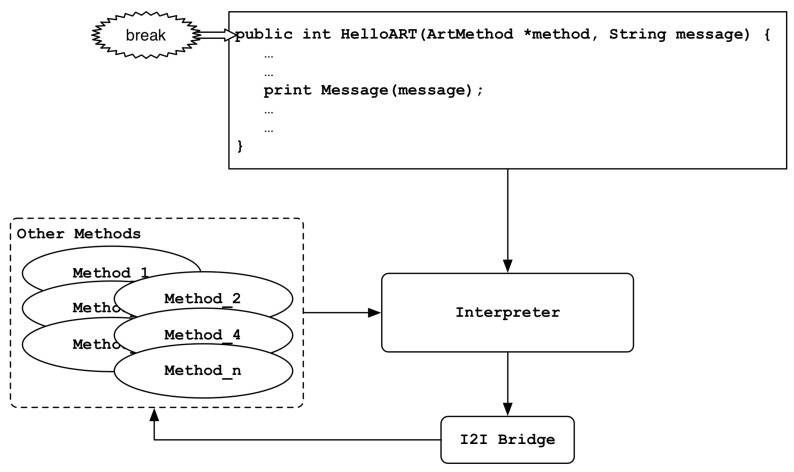
Set the event at method’s header.

**Figure 5:**
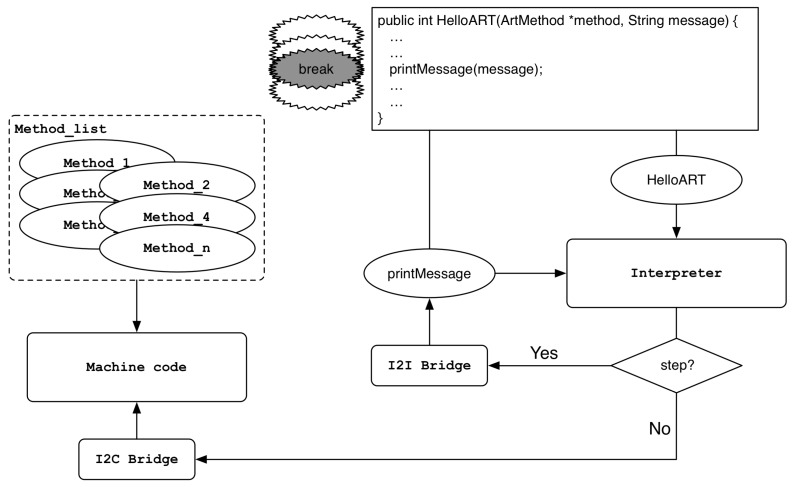
Set the event at method’s body.

kFullDeoptimization/kFullUndeoptimization: When the kFullDeoptimization request is passed to the ProcessDeoptimizationRequest() function, bridges and trampolines are set at entry points of the method so that all methods of the application operate in the interpreter domain. Conversely, in the case of a kFullUndeoptimization request, method execution through the interpreter is disabled, and bridges and trampolines are set at entry points of the method to act as the method’s original execution flow. As shown in Figure 4, the above request occurs when we break or release a breakpoint in a method’s header.
kSelectiveDeoptimization/kSelectiveUndeoptimization: kSelectiveDeoptimization is a request to execute only certain methods in the interpreter area. As shown in Figure 5, when a breakpoint is placed on or off a method body, the request is made and the entry points of the methods are set to the appropriate bridges and trampolines via the UpdateEntryPoints() function.
  Assume that there is a part in the body of Method A that calls Method B. It is important to note that Method A is executed in the interpreter area, and Method B, in the same step as step over or step into the part where Method B is called, is executed in the interpreter area. On the contrary, in case of resume, another entry point update is performed, and Method B is executed as machine code through Interpreter_To_Compiled_Code_Bridge. When the call to Method B in the body of Method A is finished, the remaining part of Method A is transferred to the interpreter area through Quick_To_Interpreter_Bridge.

### 3.2. Dynamic Analysis on Applications

**Figure 6:**
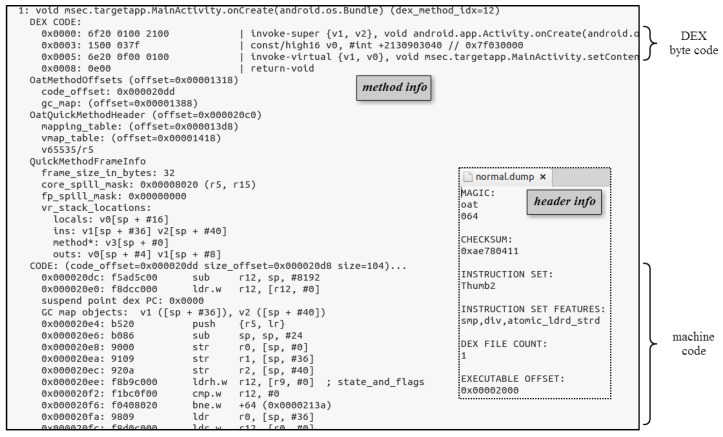
OAT file dump from oatdump.

As detailed by the analyses thus far, there is a class.dex file in the APK file, even in the ART environment, and static analysis can be performed on ART without new tools or analysis techniques. The DEX file of the application, which is the main target of static analysis, exists in the OAT file as well as in the APK file. These OAT files can be analyzed using the oatdump tool. Figure 6 shows an OAT file analysis using oatdump. The oatdump tool returns the header information of the OAT file and all class and method information in the OAT file. In the case of a method, the Dalvik bytecode and its corresponding machine code are shown.

**Figure 7:**
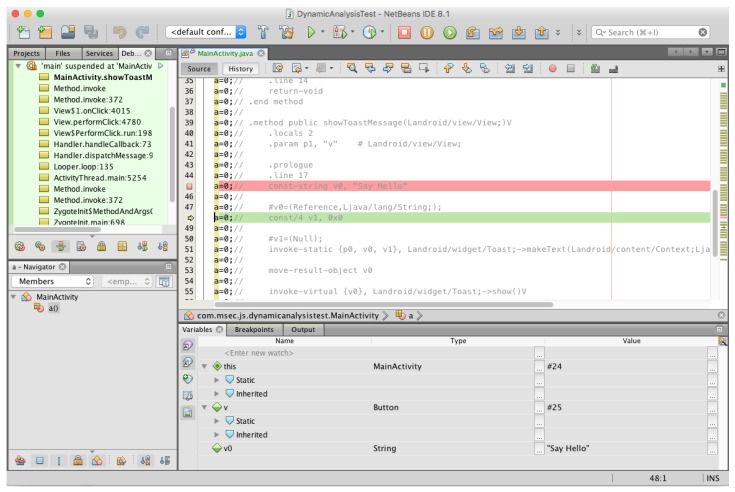
Dynamic analysis of an application using NetBeans.

Next, it can be said that dynamic analysis completely matches the existing analysis technique. ART provides application dynamic debugging via JDWP similar to the Dalvik VM. Typical Android application dynamic analysis tools include IDA and NetBeans, which provide dynamic analysis through JDWP. In dynamic analysis, if you generate an event in the method you want to analyze, the Dalvik bytecode of the method is exposed as usual, and the contents of the method are easily analyzed (see Figure 7). Thus, despite the changes in runtime system, both static and dynamic reverse engineering are possible using the same tools and techniques. The root cause of this is the exposure of the Dalvik bytecode.

**Figure 8:**
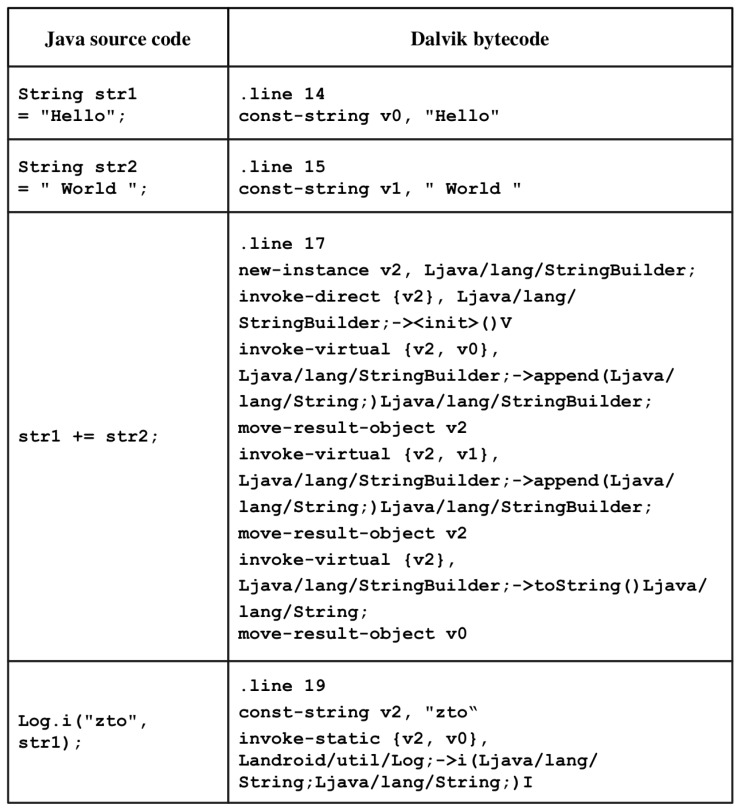
Original Java source code vs. Dalvik bytecode.

Figure 8 compares an original Java source code with its generated Dalvik bytecode. The Dalvik bytecodes corresponding to each Java source code listed on the left are clear. In the case of the assignment operator, the type of variable and its contents are immediately accessible. In the case of string concatenation, procedures omitted at the time of development are indicated, and the amount of code is increased so that there is no difficulty in its interpretation. You can also acquire information on the type of arguments to be passed to the method, its contents, class information to which the method to be called belongs, and information on method prototypes. In addition, line information and variable names in the Java source code are exposed, and detailed method information can be obtained.

On the other hand, unlike Dalvik bytecode, machine code does not provide symbol information such as strings, class names, and method names used in an application, and thus it is difficult to analyze. Therefore, in the case of application analysis through machine code, the Dalvik bytecode should be analyzed based on the signatures of the codes generated when it is compiled into machine code.

**Figure 9:**
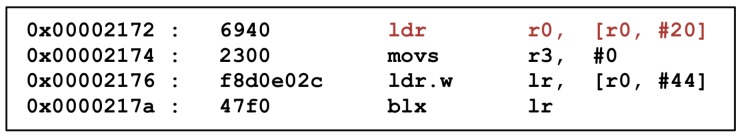
Function call signature.

The code in Figure 9 is the code signature that calls the method. The ldr r0, [r0, INDEX] command represents the index for ArtMethod in ArtMethodArray. In this case, because the method that can be used as an index includes a preloaded method, it is not possible to analyze which method is called other than the information that the method is called only by the corresponding OAT file. That is, it is quite challenging to use signature-based application analysis to check for correct information such as which method is called, what string is binding, and so on. Therefore, to protect your application from reverse engineering, it is important to minimize the exposure of the Dalvik bytecode.

## 4. Proposed Scheme

In ART, an executable OAT file contains a mix of machine code and Dalvik bytecode, and the execution flow is separated for each method to check that the application is running. Therefore, we confirmed that the existing Dalvik-based reverse engineering analysis technique can be applied by switching the artificial execution flow to Dalvik bytecode. This paper proposes a scheme to protect the application by exposing the trap code which is arbitrary fake code to the bytecode part of the OAT file. Given the structural characteristics of the ART, the Dalvik bytecode has no choice but to be exposed.

### 4.1. Principal Idea

**Figure 10:**
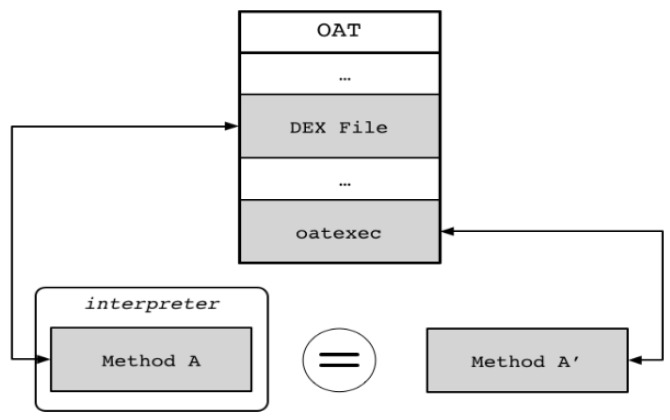
Dalvik bytecode and machine code in OAT.

**Figure 11:**
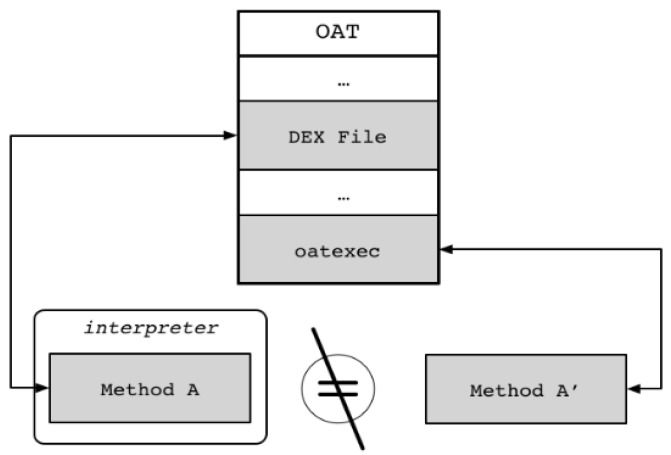
Basic idea of proposed scheme.

As detailed in the above ART analysis, one method exists in the form of Dalvik bytecode and one exists in the form of machine code in the ART environment. Assume that Method A, shown inside the interpreter of Figure 10, is in the form of Dalvik bytecode, and Method A’ is in the form of machine code. They differ in their forms, but the Dalvik bytecode is independent of the DEX file area (oatdata) of the OAT file, which is the actual executable file in ART, and machine code in the machine code area (oatexec) of the OAT file. Once the machine code is generated based on Dalvik bytecode, it is placed in an independent area, which reduces the inter-code dependency. Therefore, an attacker can easily perform reverse engineering analysis of a target application by using an existing bytecode analysis technique without analyzing the relatively difficult machine code. Thus, the basic idea of this proposed scheme is to hide the bytecode in the one-to-one mapping relationship between the bytecode and machine code in the current OAT file and to expose a bytecode that is not related to the actual machine code, as shown in Figure 11.

**Figure 12:**
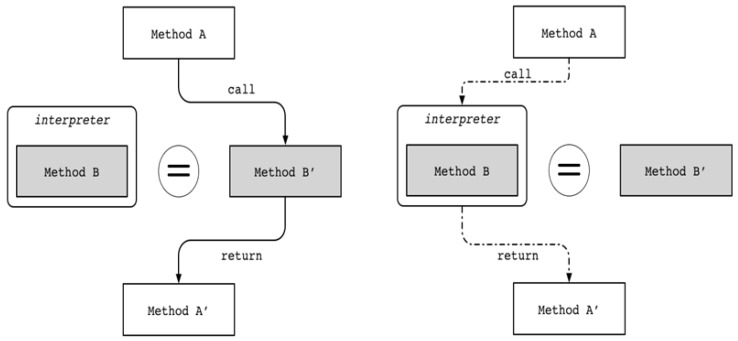
Execution flow at normal execution (**left**) and at debugging (**right**).

Use of the proposed scheme increases resistance to reverse engineering not only for static analysis but also for dynamic analysis. Dynamic debugging is accomplished through JDWP. This means that debugging using JDWP uses bytecode rather than machine code. If Method A in the form of Dalvik bytecode calls Method B (Figure 12), and the corresponding Method B’ in the form of machine code is the same as B, then the contents of Method B can be analyzed through dynamic analysis. However, when the proposed scheme is applied to the two codes through Dalvik bytecode modulation, the results of Method B’ through normal execution and of Method B through dynamic analysis are completely different, which can greatly increase the analysis difficulty. In the end, dynamic analysis, similar to static analysis, analyzes the trap code that behaves completely differently from the actual operation, which can delay the analysis time and even lead to analysis failure.

### 4.2. Design Concept

**Figure 13:**
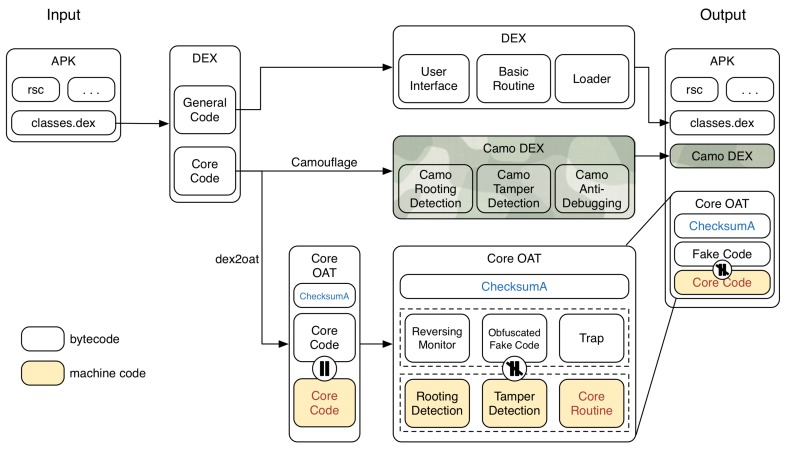
Proposed system architecture.

The proposed scheme separates the core code part to be protected from the DEX file and generates the core code as a separate OAT file, which is called the *Core OAT* file, as shown in Figure 13. Next, to confuse the analyst, a DEX file is created that pretends to be bytecode corresponding to the machine code, which is called *Camo DEX (Camouflage DEX)* in this paper.

#### 4.2.1. Core OAT Generation

Core OAT is an OAT file that contains core code in the form of machine code. Core OAT compiles the core code existing in the original DEX file through dex2oat, then modifies the oatdata section where the DEX file exists as mentioned in Section 4.1; as a result, the bytecode existing in Core OAT has modulated bytecode instead of core code. In the proposed scheme, the bytecode corresponding to rooting detection modifies with the reversing monitoring bytecode, bytecode corresponding to the tamper detection modifies with the obfuscated fake code, and the core routine modifies with the trap code having no relation to the original code to confuse the analyst’s reverse engineering process. This Core OAT file can either be included in the APK to be dynamically loaded from the local device or be distributed through a server as in [32,33,34]. The choice of distribution method may vary depending on the execution environment and security policy.

#### 4.2.2. Camo DEX Generation

Camo DEX has the same package name, class, and method structure as the actual Core OAT; however, it is a file that is configured to perform a completely different operation from Core OAT at run time. The behavior can be simply an application that prints “Hello World”, or any Android application such as a calendar or file browser. Camo DEX, however, can be said to act as a trap because it appears to be executing the code corresponding to the core routine, which is the target of the dynamically loading.

Camo Dex plays a role not only in trapping but also in solving Core OAT compatibility issues. The original OAT file has various checksum values, as shown in Table 1, based on various information from the generation process. Even if there is one identical APK file, the OAT file that has been compiled may have a different checksum due to slight differences depending on the environment of the installed device. The Core OAT distributed with the proposed scheme also has its own checksum values according to the environment at the time of generation, and problems arise when loading classes dynamically because of these values.

#### 4.2.3. Core OAT Loading and Execution

**Figure 14:**
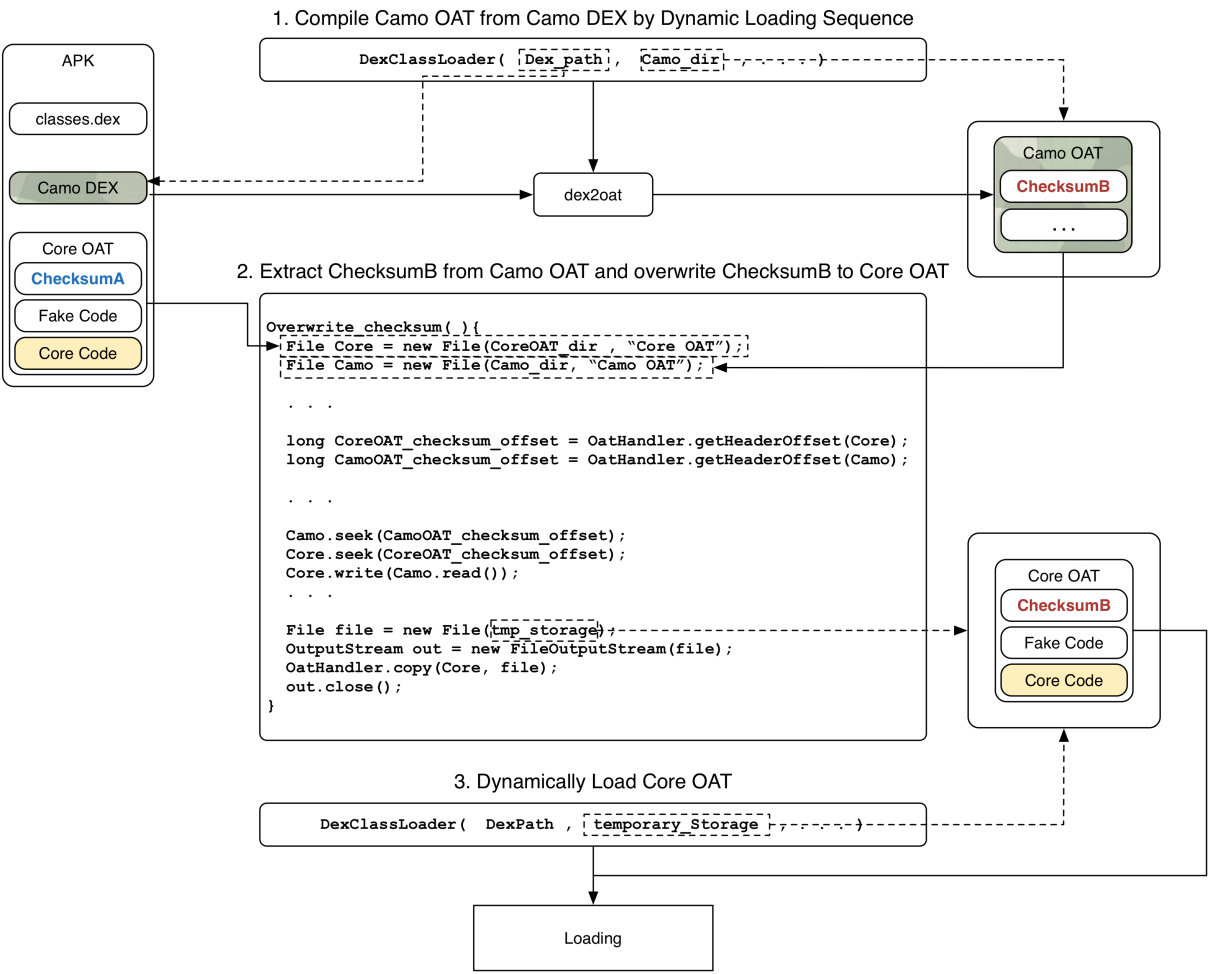
How to load Core OAT from Camo DEX.

As shown in Figure 14, an application with the proposed scheme should be able to dynamically load a class that contains a separate Core OAT . It is assumed that the Core OAT file is included in the APK. The first target for dynamic loading is Camo DEX. Assuming the cache does not exist when the application first runs, DexClassLoader uses dex2oat to compile Camo DEX into Camo OAT and store it in the cache path specified in the second parameter of DexClassLoader. From the saved Camo OAT, extract the ChecksumB value corresponding to Table 1 and overwrite the checksumA in the Core OAT with ChecksumB. At this time, delete the Camo OAT in the cache path and replace it with Core OAT . Now, Core OAT along with ChecksumB will act as Camo DEX’s cache and the application will run normally.

## 5. Experimental Results

To evaluate the proposed scheme, the experiment on reverse engineering resistance was carried out, comparing with the result of applying commercial obfuscation tools.

### 5.1. Experimental Setup

**Figure 15:**
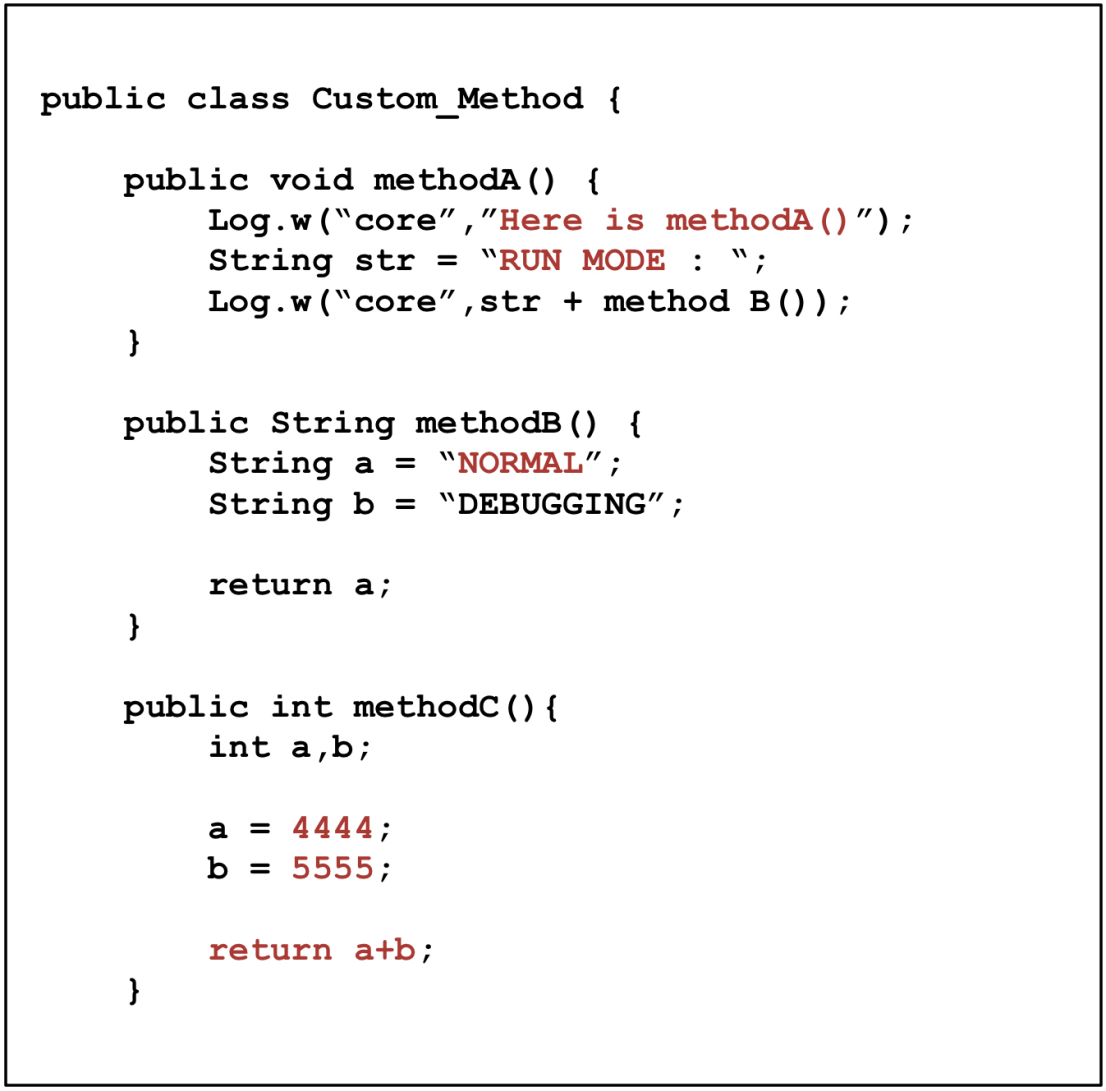
Sample source code.

We first wrote a simple code, as shown in Figure 15, and then applied ProGuard [15] and DexGuard [14], which are commercial obfuscation tools, as well as the proposed scheme. The degree of difficulty was evaluated by applying existing reverse engineering techniques to APKs independently built. The versions of ProGuard and DexGuard used in this experiment were 4.7 and 7.0.31, respectively, and the obfuscation options were set to default values for each tool. Running the APK was done on Google Nexus 5 devices with Android 5.1 Lollipop MR1, Android 6.0 Marshmallow, and Android 7.0 Nougat. Note that the specification of DexClassLoader has changed since Android 8 (API level 26), so the proposed scheme works up to Android 7 which occupies more than 60% of the Android OS market share [35].

### 5.2. Resistance to Reverse Engineering with ProGuard

**Figure 16:**
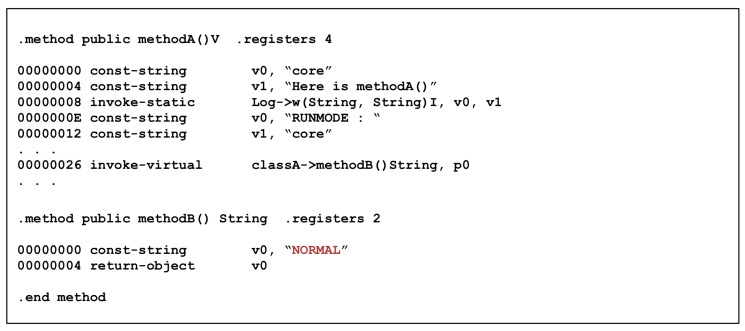
Disassembled MethodA and MethodB with ProGuard.

**Figure 17:**
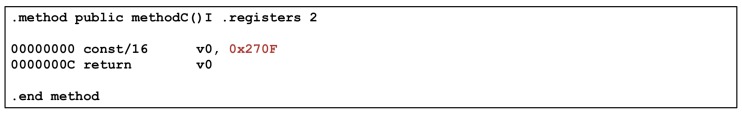
Disassembled MethodC with ProGuard.

Experiments were performed on resistance against reverse engineering attacks of obfuscated sample code through ProGuard, which is the most basic obfuscation solution. Figure 16 shows that the string data value and the routine of the function are exposed as naive. As a result of analyzing the application using ProGuard, it can be seen that the output is the same except for the identifier name and debugging information of the original code. In addition, Figure 17 is optimized for ProGuard; hence, the optimized code can be observed for easy analysis. This is why ProGuard is vulnerable to reverse engineering attacks.

### 5.3. Resistance to Reverse Engineering with DexGuard

**Figure 18:**
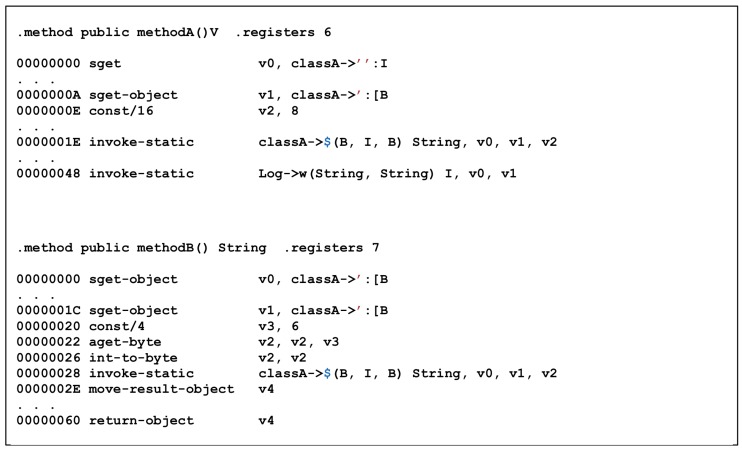
Disassembled MethodA and MethodB with DexGuard.

**Figure 19:**
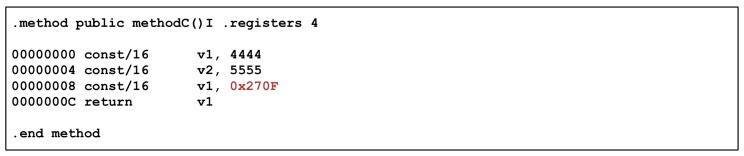
Disassembled MethodC with DexGuard.

Unlike ProGuard, DexGuard cannot obtain meaningful information only through straightforward disassembling using classA->’ object and classA->$(B,I,B) function common to both methodA() and methodB(), as shown in Figure 18. The code in Figure 19 also shows less optimization than ProGuard. However, it is a very simple routine, so there is not much applied to techniques that make analysis difficult.

**Figure 20:**
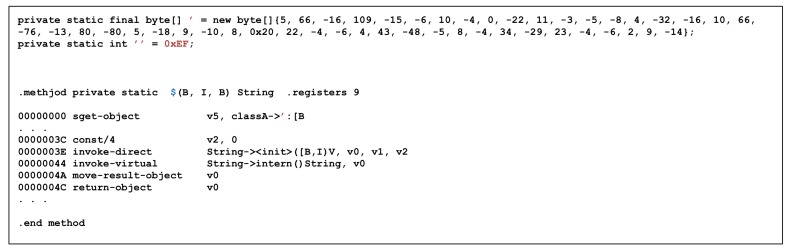
Disassembled Class->$(B,I,B).

Figure 20 is a disassemble code of classA->’ object and classA->$(B,I,B) functions. It can be observed that a String is created through a specific operation. The String generated from the $ function is used as the input value of the Log, which is generated by Figure 18. Thus, reverse engineering for DexGuard can also be achieved without significant difference from ProGuard, even though there is a certain level of difficulty in finding decryption routines and decoding the hidden data or routines.

### 5.4. Resistance to Reverse Engineering with Proposed Scheme

This section compares and evaluates reverse engineering attack resistance and performance through static and dynamic analysis of an application with the actual proposed scheme.

**Figure 21:**
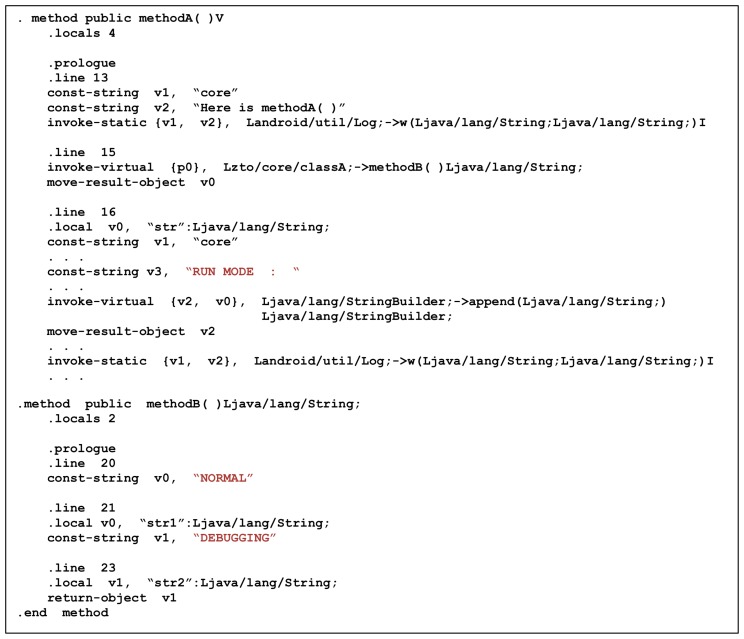
Disassembled Camo Dex.

**Figure 22:**
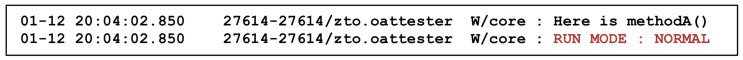
Result of Method A.

Figure 21 shows the smali code that extracted and disassembled the Camo DEX file from the application with the proposed scheme. Method A is a method that prints a string “Here is methodA ()” through the Android log method, then calls Method B and prints the returned strings by the result. Method B assigns the strings “NORMAL” and “DEBUGGING” to the v0 and v1 registers, respectively, and returns the v1 register. Therefore, when you call Method A, you can expect `RUN MODE: DEBUGGING” to appear after the string “Here is methodA ()”. However, as a result of actual execution, “NORMAL” is output instead of the string “DEBUGGING”, as shown in Figure 22.

**Figure 23:**
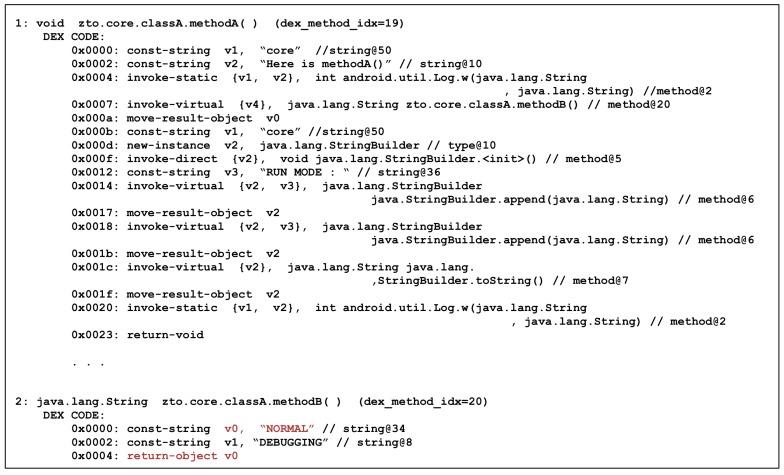
Dump Method A and Method B in Core OAT.

The above results show that the Camo DEX file is statically analyzed and the result is different from the expected result. There is no difference when you look at the contents of the OAT file that is actually executed. Figure 23 shows the dump of the OAT file using the oatdump tool. In Core OAT, you can still see that Method B returns the string “DEBUGGING” and prints it in Method A.

**Figure 24:**
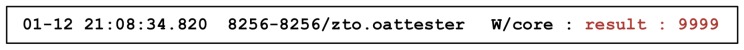
Result of Method C.

Next, the result of executing Method C is shown in Figure 24. It can be observed that Method C returns 9999 through the execution result, but, in the result of dumping the OAT file, 2222 and 1111 are allocated internally in Method C and then their difference is returned. That is, according to Dalvik bytecode, the value that Method C should return is 1111, not 9999. However, the machine code can be used to understand the results of Method C. In the machine code, you can find the sum of 5555 and 4444.

**Figure 25:**
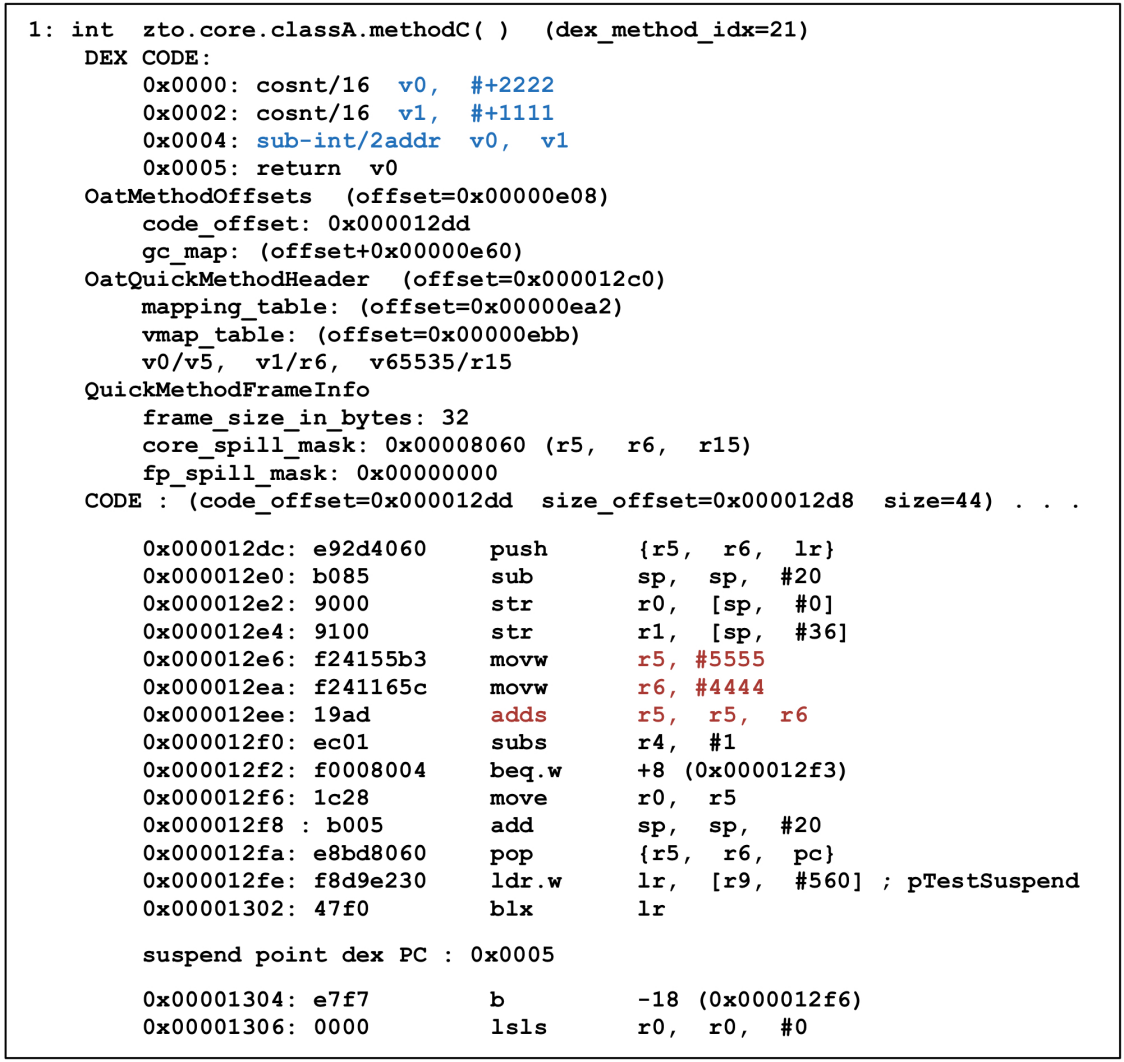
Dump Method C in Core OAT.

Thus, we can see that method analysis becomes more difficult by modulating its contents between the Dalvik bytecode and native code so that they are not the same, as shown in Figure 25. Analysis of methods based on Dalvik bytecode, which is easy to analyze, yields incorrect analysis results, and then the method must be analyzed based only on the native code. Therefore, the contents of the original Dalvik bytecode are not exposed at the time of static analysis for the application where the proposed scheme is applied, and it is possible to greatly enhance the difficulty of static analysis because analysts must analyze machine code with a high analytical difficulty.

### 5.5. Difficulty and Overhead Comparison

As is the case in the existing Dalvik environment, it is possible to analyze the application with only the Dalvik bytecode reverse engineering techniques in the ART environment. In contrast, in the case of an application using the proposed scheme, various skills such as machine code reverse engineering and runtime system analysis technology are additionally required. Therefore, the proposed scheme increase the difficulty of reverse engineering attacks. Based on the evaluation of reverse engineering attack resistance, the ability required for analysts to analyze obfuscated applications compared to existing obfuscation tools is shown in Table 2.

As the application is started, additional time and storage space are required to perform tasks related to the proposed scheme. As a result of experiments based on 17 KB Core OAT and 33 KB Camo DEX, the additional time required to execute the application was 17.793 ms, as shown in Table 3. The proposed scheme has a run-time overhead 1.7 times that of the original application. It is slower than ProGuard, but faster than DexGuard. In the case of ProGuard, a simple renaming technique is applied in the process of APK generation and optimization is performed. On the other hand, in DexGuard, the overhead is large because the decryption routine is executed each time the function is executed. The proposed scheme is expected to be suitable for practical use with less overhead than the commercial tool DexGuard.

In the end-user device environment, the storage capacity is the same size as the file stored in the application local directory. The simplest expression is 2xCamoDex+2xCoreOAT, where the minimum capacity of Camo DEX is approximately 33 KB, the most basic DEX file size.

## 6. Discussion

### 6.1. Core OAT Dynamic Loading Requirement

**Figure 26:**
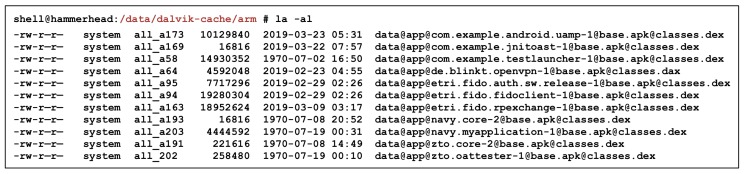
OAT file creation path on Android device.

In this proposed scheme, the Core OAT file containing the core code is designed to be loaded dynamically. The reason for this dynamic loading is the root privilege issue and OAT file modification problem at the time of installation. The application is installed as an OAT file on the Android device. As shown in Figure 26, the generated OAT file is in the “/data/dalvik-cache/[arch]/” path. The partition requires a root privilege and cannot be accessed by end users or general applications. A permission problem occurs when attempting to apply the proposed scheme to the OAT file created after the installation process.

Therefore, if it is difficult to change the created OAT file, it is necessary to intervene in the OAT file creation to apply the required code splitting, which can be done by artificially modifying the Android platform. For this reason, the OAT file applied by the proposed scheme is designed to be recognized as a normal cache file corresponding to the DEX file through class dynamic loading.

### 6.2. Additional Trapping through Core OAT Modulation

Core OAT files require basic understanding of OAT file structure and DEX file structure to modulate the OAT file, and the degree of trap implementation may vary depending on the understanding of the Dalvik bytecode command. The object to be modulated is Dalvik bytecode, and Dalvik bytecode exists in the DEX file located in the oatdata section of the OAT file. The code_item entry in the DEX file contains the actual register information and the actual Dalvik bytecode used by the method. Generally, the modulation process can be divided into two types.

**Figure 27:**
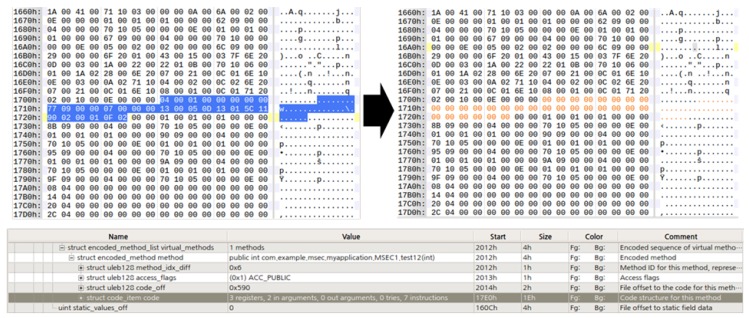
Delete the Dalvik bytecode.

Figure 27 shows that all code_item entries in the method have been replaced with zeroes. In this case, the register information and access information of the method are all 0, and all commands are shown as a NOP state. This method has the advantage of being able to completely block exposure of the original Dalvik bytecode from future reversing and is simple to implement. However, it has the disadvantage of being very noticeable.

**Figure 28:**
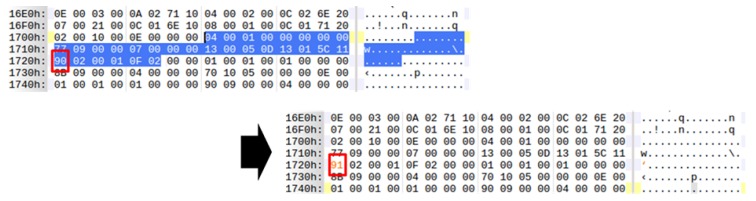
Dalvik bytecode partial modulation.

To compensate, only a part of the Dalvik bytecode can be modulated. Figure 28 shows altering the opcode of the instruction by modifying the “add-int” Dalvik bytecode command value 0x90 with the “sub-int” Dalvik bytecode command value 0x91. Simply by increasing the value of the opcode by 1, the method’s Dalvik bytecode becomes completely different from the machine code. In addition, operands can be further modified to provide more complexity here and can be replaced by completely different opcodes. This approach can intentionally expose the fake Dalvik bytecode to the analysts. It can confuse the analyst more effectively and force Dalvik bytecode and machine code to compare and contrast.

The above procedure allows for the setting of various traps by effectively modulating the original Dalvik bytecode with a primary trapping operation. The modified Core OAT can be deployed through the server or distributed within the APK, and later applications can use the original Core OAT through class dynamic loading on the Android device.

### 6.3. Core OAT Integrity Check

In the proposed scheme, the checksum of Core OAT is changed to the checksum value of Camo OAT, and so integrity verification may become vulnerable. In this case, the Core OAT may be replaced by the malicious OAT, or the application protection routine on the Core OAT may be disabled by the analyst. Additional authentication mechanisms [36,37,38] are needed to address these vulnerabilities. Applications could use only authenticated OAT files using a challenge-response method that allows the application and Core OAT to communicate with and authenticate each other rather than a simple file verification method. If the file is not an authenticated OAT file, the Core OAT should be reacquired.

### 6.4. Limitations

The proposed scheme interferes with the analysis by exposing the trapping code instead of the core routine. Furthermore, several machine code reversing skills are required to analyze the code generated by the proposed scheme. Unlike the existing solutions, which are easily reversible only with static analysis, the proposed scheme increases the analysis difficulty by requiring knowledge of ART structure and machine code analysis skills on Android architecture. However, if the attacker is able to analyze the machine code of the Core OAT based on an understanding of the machine code and with knowledge of the ART system structure, the proposed scheme also fails. Compared to other commercial obfuscation solutions, the proposed scheme can compensate for these limitations because it requires relatively low execution overhead. In addition, existing obfuscation tools must use a combination of several options that require encryption, which can cause runtime overhead to be too high to be practical. However, since the proposed scheme does not require encryption, it is more advantageous to combine it with other schemes as well as has higher performance.

## 7. Conclusions

This paper describes an analysis of the contents of ART, the new runtime system of Android. Based on this analysis, we propose a reverse engineering analysis prevention technique using the relationship between Dalvik bytecode and machine code. The proposed scheme protects the application from static analysis by preventing exposure of the Dalvik bytecode, which is easier to analyze compared with machine code, and also prevents dynamic analysis using ART’s execution transition structure. In addition, the proposed scheme introduces Core OAT and Camo DEX to provide versatility and practicality. Camo DEX can also improve the reversing resistance of the proposed scheme by acting as an additional trap along with solving the original Core OAT compatibility problem.

The reverse engineering resistance of the proposed scheme is demonstrated through reverse engineering analysis experiments on its application. In the reverse engineering analysis results, the original state Dalvik bytecode could not be obtained by either the static analysis or dynamic analysis. It was confirmed that only the trap code, which operates completely differently, was exposed.

In conclusion, the proposed anti-reversing scheme can be used as a core technology to protect newly introduced ART-based Android applications from reversing by intentionally exposing the trap to the application and blocking exposure of the core code. Ultimately, it is expected to contribute greatly to the security of IoT devices that are compatible with ART-based mobile devices.

## Figures and Tables

**Table 1:** List of OAT file checksum data.

Sectioname	Section Component Name
OatHeader	alder32_checksum
OatHeader	image file location oat checksum
OatHeader	image file location oat data begin
OatDexFile	dex file location checksum
Dex File	dex file checksum
Dex File	SHA1 signature

**Table 2:** Required reversing skills to analyze protected code.

Target Objects	Required Skills	ProGuard	DexGuard	Proposed Scheme
	Decompile	✓	✓	✓
	Dalvik bytecode	✓	✓	✓
Bytecode	API knowledge	✓	✓	✓
	Repackaging	✓	✓	✓
	Dalvik VM & ART framework			✓
Crypto routine	Encryption algorithms		✓	
	CPU instruction			✓
Machine code	Disassemble			✓
	Debugging			✓
	OAT(ELF) file structure			✓

**Table 3:** Runtime overhead.

	Original	ProGuard	DexGuard	Proposed Scheme
Execution time (milliseconds)	11.494	11.073	58.699	17.793
